# Advancements in dendritic cell vaccination: enhancing efficacy and optimizing combinatorial strategies for the treatment of glioblastoma

**DOI:** 10.3389/fneur.2023.1271822

**Published:** 2023-10-31

**Authors:** Robert C. Subtirelu, Eric M. Teichner, Arjun Ashok, Chitra Parikh, Sahithi Talasila, Irina-Mihaela Matache, Ahab G. Alnemri, Victoria Anderson, Osmaan Shahid, Sricharvi Mannam, Andrew Lee, Thomas Werner, Mona-Elisabeth Revheim, Abass Alavi

**Affiliations:** ^1^Department of Radiology, Hospital of the University of Pennsylvania, Philadelphia, PA, United States; ^2^Sidney Kimmel Medical College, Thomas Jefferson University, Philadelphia, PA, United States; ^3^Department of Physiology, Faculty of Medicine, Carol Davila University of Medicine and Pharmacy, Bucharest, Romania; ^4^Division of Technology and Innovation, Oslo University Hospital, Oslo, Norway; ^5^Faculty of Medicine, Institute of Clinical Medicine, University of Oslo, Oslo, Norway

**Keywords:** glioblastoma, dendritic cell vaccination, positron emission tomography, antigen loading, immunotherapy

## Abstract

Glioblastomas (GBM) are highly invasive, malignant primary brain tumors. The overall prognosis is poor, and management of GBMs remains a formidable challenge, necessitating novel therapeutic strategies such as dendritic cell vaccinations (DCVs). While many early clinical trials demonstrate an induction of an antitumoral immune response, outcomes are mixed and dependent on numerous factors that vary between trials. Optimization of DCVs is essential; the selection of GBM-specific antigens and the utilization of ^18^F-fludeoxyglucose Positron Emission Tomography (FDG-PET) may add significant value and ultimately improve outcomes for patients undergoing treatment for glioblastoma. This review provides an overview of the mechanism of DCV, assesses previous clinical trials, and discusses future strategies for the integration of DCV into glioblastoma treatment protocols. To conclude, the review discusses challenges associated with the use of DCVs and highlights the potential of integrating DCV with standard therapies.

## 1. Introduction

Glioblastoma, or “glioblastoma multiforme” (GBM), is the most common subtype of diffuse gliomas (1). According to the World Health Organization (WHO) classification of adult-type diffuse gliomas, these tumors are categorized into two major classes based on the mutation status of isocitrate dehydrogenase 1 and 2 (IDH1 and IDH2). GBM, which is IDH-wildtype, is graded as CNS WHO grade 4 and has the worst prognosis. Histological diagnosis of glioblastoma is defined by the presence of necrosis with or without cellular pseudopalisading and/or microvascular proliferation. Other histological features include pleomorphic cells, mitotic activity, and intravascular microthrombi. Glioblastoma can be further divided into two subtypes: primary and secondary. Primary glioblastoma, the more prevalent subtype, arises *de novo*, without evidence of a precursor lesion. Secondary glioblastoma arises from pre-existing, lower-grade astrocytomas (2).

The current standard treatment for glioblastoma consists of maximal surgical resection, followed by radiation therapy with concurrent and adjuvant temozolomide, known as the Stupp regimen (3). This approach has demonstrated minimal improvement in survival, with a median progression-free survival (PFS) of 7.8 months, a median overall survival of 14.6 months, and a 5-year survival rate of under 10% (2).

Given their promising results in extending survival times in patients with other types of cancer, dendritic cell vaccinations (DCVs) have been explored for their immunotherapeutic potential in treating glioblastoma. Dendritic cells are specialized antigen-presenting cells that acquire and process antigens, migrate to lymph nodes, and activate T cells, thus inducing protective immune responses. Previous studies have shown that DC vaccines can safely induce long-lasting antitumor immune responses with minimal or no toxic effects (4). For instance, the vaccine sipuleucel-T extends median survival times by 4 months in patients with prostate cancer (5). Cho et al. demonstrated that adjuvant immunotherapy with whole-cell lysate dendritic cell vaccination may improve short-term survival in patients with glioblastoma, with significantly higher 1-, 2-, and 3-year survival rates as well as PFS in comparison to a control group (6). Batich et al. conducted three separate clinical trials over a decade, using cytomegalovirus (CMV)-specific dendritic cell vaccines in patients with newly diagnosed glioblastoma; about one-third of these patients exhibited no tumor recurrence 5 years post-diagnosis, despite challenges in optimizing vaccine dosage and antigens (7).

## 2. Mechanism and background

DCs, which serve as antigen-presenting cells, play a pivotal role in the immune system, both by facilitating tolerance to avert T-cell mediated host attacks, and by stimulating adaptive immune responses. In the absence of infection, DCs persistently present self-antigens to T cells, thereby fostering the development of regulatory T cells (Tregs). This process establishes tolerance and inhibits immune responses against the host and harmless environmental antigens, which cannot induce immunoactive responses in the human body (8). Following the onset of infection, DCs process and present antigens to T cells, bolstering the production of helper and effector T cells, ensuring effective communication between the innate and adaptive immune systems (9, 10).

Given DCs’ efficacy in facilitating T-cell activation, which is essential for anti-tumor immunity, these antigen-presenting cells serve a central role in bridging the gap between tumor recognition and T-cell mediated tumor elimination. Extensive research has therefore culminated in the development of DCVs as active immunotherapies (11).

### 2.1. DCV production methodology

**Figure 1:**
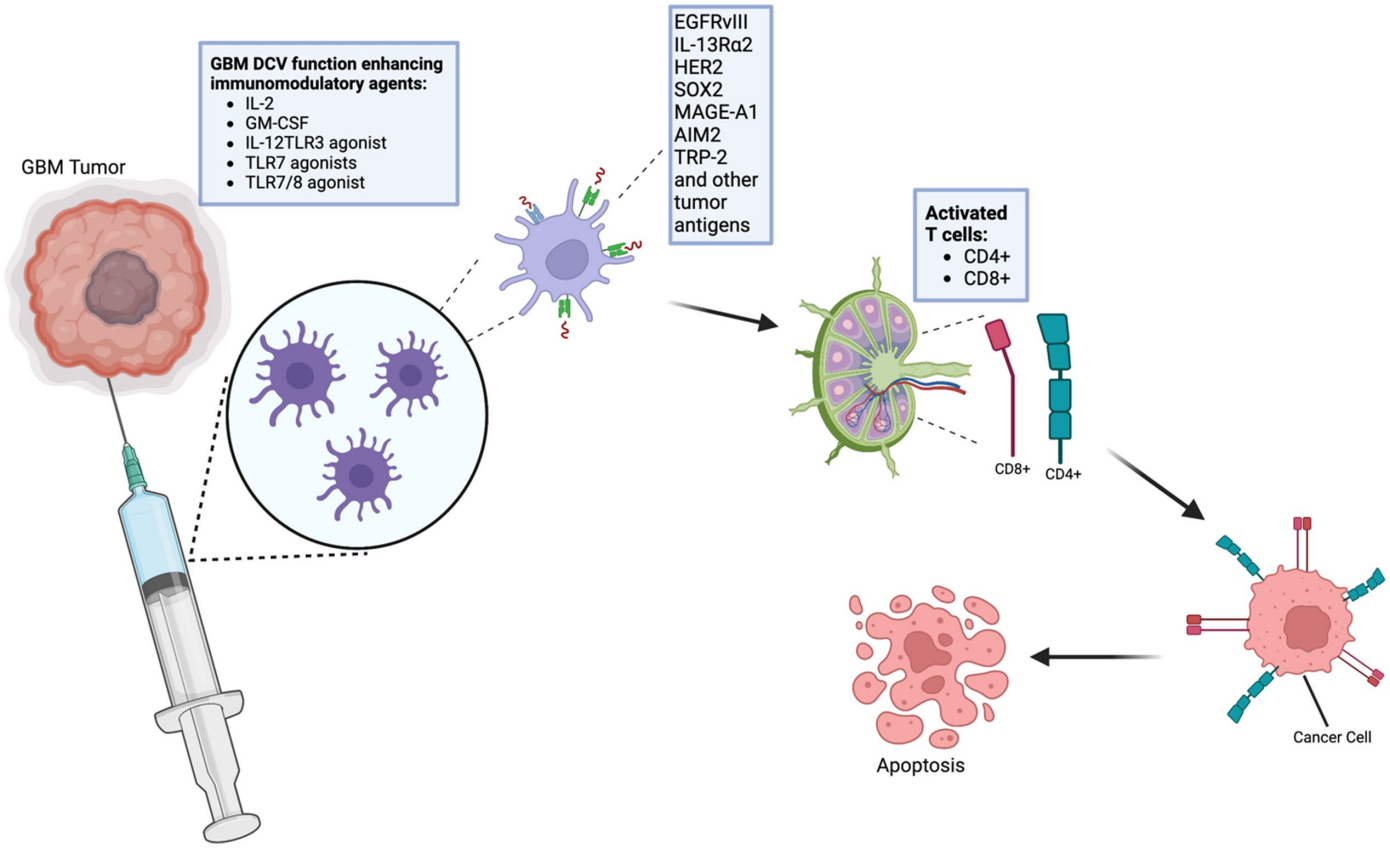
Schematic representation of the process of dendritic cell vaccinations (DCVs). DCVs have certain antigens that induce immune responses against cancer, including glioblastomas. Certain agents can improve the efficiency of the vaccines against glioblastoma. This image was created using BioRender.com. IL-2, Interleukin 2; GM-CSF, Granulocyte-Macrophage Colony-Stimulating Factor; IL-12, Interleukin 12; TLR3, Toll-Like Receptor 3; TLR7, Toll-Like Receptor 7; TLR7/8 agonist, Toll-Like Receptor 7/8; EGFRvIII, Epidermal Growth Factor Receptor variant III; IL-13Ra2, Interleukin-13 Receptor Alpha 2; HER2, Human Epidermal Growth Factor Receptor 2; SOX2, Sex-Determining Region Y-Box 2; MAGE-A1, Melanoma Antigen Gene A-1; AIM2, Absent in Melanoma 2; TRP-2, Tyrosinase-Related Protein 2.

**Figure 2:**
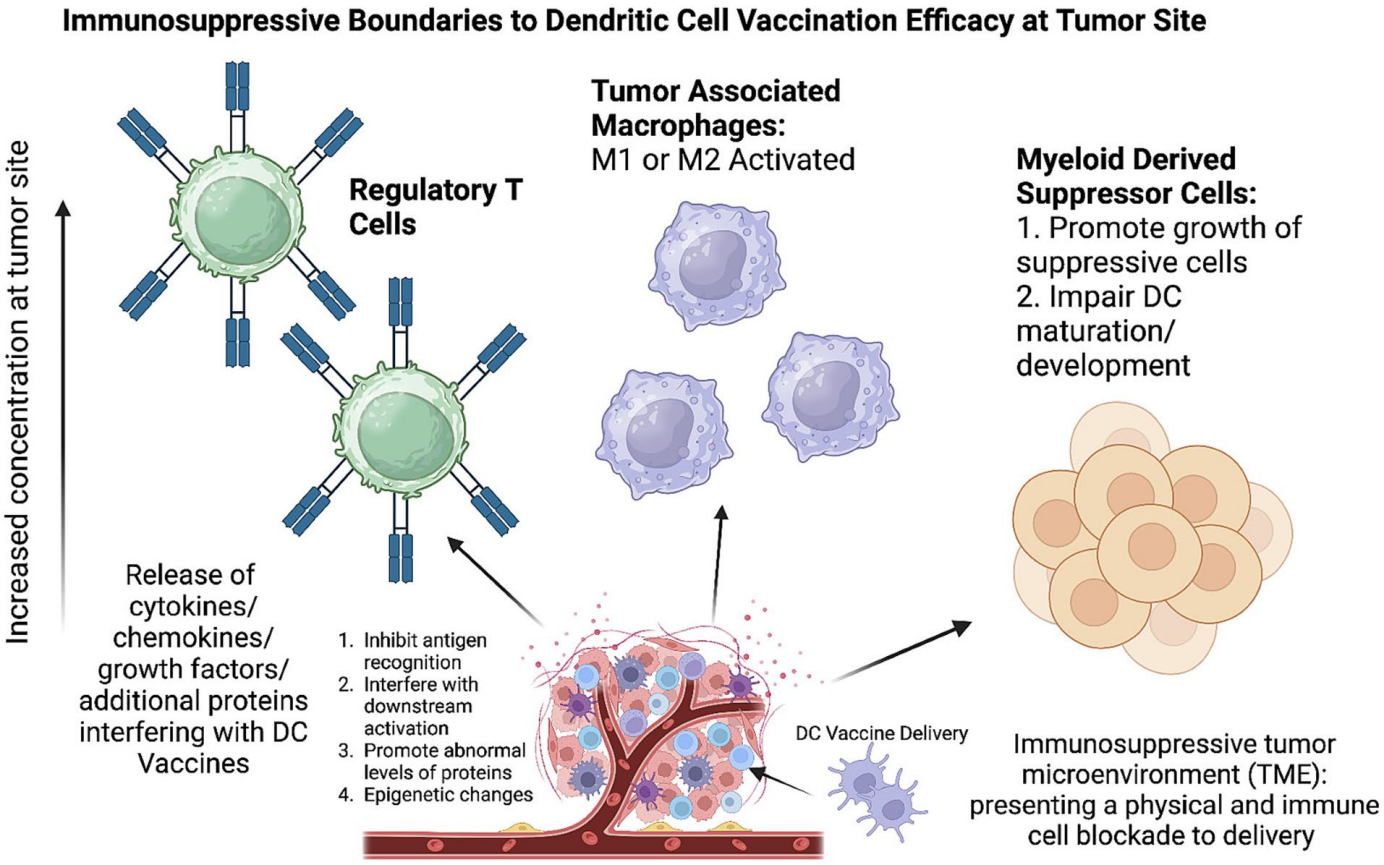
Immunosuppressive tumor microenvironment (TME) inhibits effectiveness of dendritic cell vaccination. TME growth results in abnormal concentrations of immunosuppressive cells including regulatory T cells, tumor associated macrophages, and myeloid derived suppressor cells. Created with Biorender.com.

The production of DCV entails a series of critical steps:

1. Extraction: DCs are typically harvested from the patient’s blood.
2. Culturing: Once extracted, DCs are cultured *ex vivo* with a variety of cytokines, including growth factors such as granulocyte-macrophage colony-stimulating factor (GM-CSF) and interleukin IL-4.
3. Loading with Antigens: These cultured cells are then loaded or “pulsed” with tumor-specific antigens. The introduction of these antigens is critical as it allows the DCs to present these specific markers to the immune system, enhancing the specificity and efficiency of the vaccine.
4. Activation and Reintroduction: Upon activation, the DCs are reintroduced into the patient’s body via intravenous or intradermal routes. They then travel to the tumor microenvironment or lymph nodes.
5. Antigen Presentation and Immune Activation: In the targeted regions, the DCs present the antigen to CD4+ and CD8+ T cells, thereby activating both humoral and cell-mediated immune responses (12, 13) (Figures 1, 2).

The specificity of the tumor antigens used in the loading step holds paramount importance. These antigens ensure that the resulting immune response is tailored to target and combat the tumor cells specifically, improving the efficiency and potential efficacy of the DCVs. Their distinct advantage lies in their capacity to enhance anti-tumor responses early on, and their potential to target a broad spectrum of tumor-associated antigens more effectively (14).

The study of DCVs spans over two decades, with the first clinical trial reported in 1996 for the treatment of follicular B-cell lymphoma (15). Each of the four patients who received the vaccine exhibited measurable anti-tumor immune responses, from partial tumor regression to total resolution of all disease evidence. In a 2006 phase III clinical trial involving patients with hormone-refractory prostate cancer, the median overall survival was markedly higher in the group receiving DCVs compared to the placebo group. This breakthrough led to the development of Sipuleucel-T (Provenge), the first and only approved DCV for prostate cancer (16, 17).

In the context of glioblastoma, the first utilization of DCVs for individual patient treatment was reported by Liau et al. (17). The patient tolerated the vaccine well and exhibited a measurable cellular immune response, characterized by heightened T-cell infiltration in the tumor, despite continued tumor progression and the patient’s subsequent death several months later (18). The treatment demonstrated the potential of DCVs to elicit antigen-specific immunity in patients afflicted with GBM, underscoring a new paradigm in personalized immunotherapeutic strategies.

## 3. Clinical trials

Numerous studies, including several randomized clinical trials across various phases, have been published on the treatment of new and recurrent GBM with DCV in adults, children, and adolescents. Phase I trials, of which at least 12 were published between 2001 and 2010, have provided preliminary evidence of DCV therapy’s efficacy in treating GBM (18). For instance, Liau et al. reported the results of a Phase I trial of 12 GBM patients (seven newly diagnosed, five with recurrent disease) treated with DCV. These patients had a median overall survival (mOS) of 23.4 months, compared to a mOS of 18.3 months in a set of historical controls (19). However, other studies published during this period did not report similar success levels with DCV for GBM or other high-grade gliomas (18). Despite mixed outcomes, the foundation was laid for further advancements in DCV technology and its potential therapeutic role.

A key finding from early DCV therapy investigations is its limited toxicity. Severe side effects (grades 3–4) are rare, with few cases across many trials, not all necessarily attributable to the vaccine (16). In a study conducted by Mitchell et al., a type 1 hypersensitivity reaction was observed in a GBM patient following intradermal administration of a DCV formulated with granulocyte-macrophage colony-stimulating factor (GM-CSF) (20). Other severe side effects include seizures and one case of peritumoral edema (21). In contrast, more frequent were grade 2 or lower side effects, which include injection-site reactions, flu-like symptoms, or meningeal irritation. However, these symptoms are also observed with other GBM therapies or may be attributable to the disease course itself. Overall, patients with GBM generally tolerate DCV therapy well, even in cases of advanced disease (16).

Recent randomized phase II clinical trials have further established that DCV can confer survival benefits to GBM patients. For example, Cho et al. reported a significant increase in mOS (31.9 months vs. 15.0 months) as well as median progression-free survival (mPFS) (8.5 months vs. 8.0 months) for newly diagnosed GBM patients when comparing vaccinated patients to controls (6). Similarly, Jie et al. reported an mOS of 17 months for vaccinated patients compared to 10.5 months for control patients in the context of newly diagnosed GBM (22). In a study conducted by Yao et al., a total of 43 GBM patients were analyzed. Post-surgery, patients were randomized; 22 received the DCV treatment loaded with glioblastoma stem cell-like (GSC) antigens, and 21 were administered a normal saline placebo. When stratifying the data based on molecular markers, Yao et al. identified a noteworthy extension in OS to 13.7 months, up from 10.7 months, particularly in IDH1 wild type (WT) TERTMT patients. Furthermore, patients with low B7-H4 expression also showed significant prolongation in OS after the DCV treatment. Additionally, the PFS for the DCV-treated group was 7.7 months as opposed to the 6.9 months in the placebo cohort (23). Moreover, Batich et al. combined data from multiple trials to demonstrate increases in mOS of patients with newly diagnosed GBM when receiving DCV compared to controls (7, 24, 25). The results of these trials robustly support the continued investigation and development of DCV as a treatment for both newly diagnosed and recurrent GBM.

Recently, in 2023, Liau et al. revealed the results from a large Phase III trial (NCT00045968). The study treated 331 patients with both newly diagnosed and recurrent GBM, comparing a placebo group receiving only standard-of-care (SOC) medical treatment with temozolomide to an experimental group that additionally received the DCV DCVax^®^-L. Significant increases in mOS were reported in both new GBM patients (19.3 months vs. 16.5 months) and recurrent GBM patients (13.2 months vs. 7.8 months) when receiving DCV and SOC compared to SOC alone (26). The crossover design of the study necessitated the use of external controls for statistical analysis. Nevertheless, these results offer promising support for the use of DCV as an adjunct to temozolomide chemotherapy.

However, other randomized phase II clinical trials have not shown similar survival benefits for GBM patients receiving DCV. Wen et al. (NCT01280552) reported no statistically significant increases in mOS (17 months vs. 15 months) in newly diagnosed GBM patients receiving the experimental DCV ICT-107 compared to controls, though PFS was significantly but modestly increased in vaccinated patients (11.2 months vs. 9 months) (27). Furthermore, Buchroithner et al. (NCT01213407) found no significant differences between newly diagnosed GBM patients receiving the Audencel DCV and control therapy in mOS (18.8 months vs. 18.9 months) or in PFS (28.4% vs. 24.5% at 12 months) (28).

One possible explanation for the inconsistent results could be the heterogeneity of DCV products used in the trials. De Vleeschouwer, reflecting on the ICT-107 DCV trial by Wen et al., noted that the lack of consensus on the “optimal DC product” inherently reduces the generalizability of conclusions drawn from studies using any particular product (25, 27). Buchroithner et al. discussed the potential impact of dendritic cell maturation as an explanation for their results, noting that their DC maturation protocol uniquely included lipopolysaccharides/interferon gamma (IFNγ/LPS), unlike studies that demonstrated a survival benefit (26). It is interesting to note that of the studies discussed here, the ICT-107 DCV trial and the Audencel trial both matured dendritic cells using IFNγ/LPS and both failed to find increased median overall survival (25, 26). A combination IFNγ/LPS stimulus has been noted to produce mixed effects, simultaneously causing an IL-12 and cytotoxic T-lymphocyte response, while also inducing the immunosuppressive molecule indoleamine-2,3-dioxygenase (IDO) (14). Aforementioned randomized phase II trials using TNFα, IL-β, and PGE2 by Jie et al. or TNFα, IL-β, IL-6, and PGE2 by Batich et al. reported increases in median overall survival (6, 20). TNFα and PGE2 have been historically favored for DC maturation; however, PGE2 has also been shown to also induce IDO, making it difficult to tell how much of the differences in clinical benefit shown in these four studies can be attributed to differences in DC maturation stimuli (14). Additionally, Cho et al. and Yao et al. showed clinical benefit in randomized phase II trials using immature dendritic cells, with no additional maturation stimulus after culturing with GM-CSF and IL-4 (5, 21).

Another key differentiating factor between DCV products are the target antigens; the specificity and efficacy of the vaccine depends on targeting tumor-associated antigens (TAA) in tumor cells. Dendritic cells are pulsed with these antigens during vaccine production. Commonly, whole-tumor cell sources of TAAs have been used to pulse DCs such as tumor lysates in the successful trials by Cho et al. (6) and Jie et al. (22). Glioma stem cell lysate was used successfully as well in the trials by Yao et al. and the phase III trial by Liau et al. (23, 26). Whole-tumor sources contain a large set of antigenic targets which will likely include multiple TAAs, reducing the risk of a TAA-loss variant that can evade immune response induced by the vaccine. There may be additional signaling molecules in whole-tumor sources which, through mechanisms not yet elucidated, help guide the T-lymphocyte response to the tumor (16). However, Buchroithner et al. failed to find clinical benefit using tumor lysate-pulsed DCs (28). Since the vast majority of proteins in whole-tumor cell sources are benign and even nonspecific to brain tissue, it is possible that the concentration of tumor-specific and immunogenic proteins in the lysate used in this study was too low to induce a sufficient immune response and confer clinical benefit. An alternative to whole-tumor sources are molecularly-defined TAAs, where dendritic cells have been transfected with the mRNA of a specific target antigen. Molecularly-defined TAAs are more defined, specific, and consistent, and DCs may be able to be transfected with a higher load of immunogenic TAAs than is possible with whole-tumor sources (16). Molecularly-defined TAAs also allow immune monitoring of the response to specific antigens (29). Batich et al. demonstrated clinical success using CMVpp65 mRNA-transfected DCs; CMVpp65 is likely present in the majority of GBM patients but not in normal brain tissue (7, 24, 25). The ICT-107 trial used six well-known GBM TAAs - MAGE-1, AIM-2, HER-2, TRP-2, gp100, and IL-13Ra2—likely to strike a balance between avoiding immune evasion by tumor variants while maintaining as specific targeting as possible. However, no benefit was shown to median overall survival (27, 29).

Additionally, non-standardized DCV administration protocols may have contributed to the observed discrepancy in results. Aarntzen et al. demonstrated that an excessive DC concentration in the injected volume reduces overall DC migration to lymph nodes, leading de Vleeschouwer to question whether the DC concentration used in the ICT-107 trial could have been reduced to improve efficacy (27, 29, 30). In this trial, a dose of 11 × 10^6^ DCs/vaccine was used (27). Three aforementioned successful trials used smaller doses, all in the range of 1–6 × 10^6^ DCs/vaccine (22, 23, 26). However, trials by Batich et al. and Cho et al. used larger doses of 20 × 10^6^ and 20–50 × 10^6^ DCs/vaccine, respectively, with demonstrated clinical benefit; Batich et al. also used the same intradermal site of administration as the ICT-107 trial (6, 7). The lack of clear connection between dose and efficacy and the heterogeneity of vaccine products and administration methods makes a dose–response relationship unable to be characterized with current investigation. A similar difficulty exists when attempting to optimize the site of administration. Intranodal administration should theoretically maximize the quantity of DCs that are able to migrate to lymph nodes and activate immune responses, but Buchroithner et al. was not able to find success with this method (28), while the trials that showed clinical benefit used subcutaneous (6, 7) or intradermal techniques (22, 23, 26). No clear pattern emerges when comparing vaccination schedule or quantity between these trials, and it is generally unknown whether increasing the quantity or frequency of vaccine doses improves outcomes (16). Moving forward, the optimization of dendritic cell doses, administration sites, and administration schedules is necessary for the generalizability of clinical trial results.

Another challenge for DCV therapy is eliciting an antitumoral immune response amid stark immunosuppression, possibly due to concurrent antitumor therapy or the immunosuppressive tumor microenvironment of GBM. Patient immune response heterogeneity must also be considered when analyzing trial outcomes. For example, a phase II study by Wheeler et al. treated newly diagnosed and recurrent GBM patients with DCV and stratified patients into vaccine responder and non-responder statuses based on pre- and post-treatment IFNγ levels. Vaccine responders experienced significantly longer mOS (21.1 months) compared to non-responders (14.1 months) (31). Yao et al. only demonstrated clinical benefit from DC vaccination after stratifying based on isocitrate dehydrogenase 1 (IDH1) and telomerase reverse transcriptase (TERT) promoter mutations; patients with wild-type IDH1 and mutated TERT promoters showed significantly improved mOS. Additionally, Yao et al. demonstrated better responses to DCV therapy in GBM patients with lower levels of B7-H4, a CD4+ T-cell suppressor molecule (23, 32). Other immune status markers, such as the programmed cell death 1 (PD-1)+:CD8+ ratio, regulatory T-cell levels, MGMT methylation status, and cytotoxic T-lymphocyte-associated antigen (CTLA)-4 expression response to DCV, further indicate that DCV may be more efficacious in certain immunophenotypes (33–35). Integrating these markers into trial design could further refine the patient population for DCV therapy, although it may limit external validity and contribute to difficulty in comparing trial results. The ICT-107 vaccine was conceived only for GBM patients of the HLA-A1 and HLA-A2 haplotypes, which represents about 2/3rds of the Caucasian population, and de Vleeschouwer notes that this seriously limits interpretation of the results of this trial (27, 29). Future investigation should continue to identify patients that may be more responsive to DCV therapy and optimize specific DCV products and protocol for these immunophenotypes (Table 1).

**Table 1:** Randomized controlled trials of DCV therapy for GBM.

Trial	Phase	GBM	Control	TAA	Maturation	Site	Schedule|Dosing	mOS (m)^†^	PFS (m)^†^
Cho et al. (6)	II	18 (18 nd)	16	Tumor lysate		SC	4x weekly +2x biweekly +4x monthly|20–50 × 10^6^ cells	**31.9**	**8.5**
Jie et al. (22)	II	13 (13 nd)	12	Tumor lysate	TNFα, IL-1β, PGE2	SC	2x weekly +2x biweekly|6 × 10^6^ cells	**17**	
Yao et al. (23) NCT01567202	II	22 (13 nd)	21	Glioma stem cell lysate		ID	3x weekly|2–3 × 10^6^ cells	**13.7**^1^	**7.7**^1^
Buchroithner et al. (28) (Audencel) NCT01213407	II	34 (34 nd)	42	Tumor lysate	IFNγ, LPS	IN	4x weekly +5x monthly + every 3 months up to 15 doses total|1–5 × 10^6^ cells	18.8	
Wen et al. (27) (ICT-107) NCT01280552	II	81 (81 nd)	43	MAGE-1, AIM-2, HER-2, TRP-2, gp100, and IL-13Ra2	IFNγ, LPS	ID	4 weekly +4 monthly + every 6 months until tumor progression|11 × 10^6^ cells	17	**11.2**
Batich et al. (7) NCT00639639NCT02366728	II	23 (23 nd)	6	CMVpp65 mRNA	TNFα, IL-1β, PGE2	ID	3x biweekly then monthly until tumor progression|20 × 10^6^ cells	**41.1 (GM-CSF), 41.4 (Td)**	
Liau et al. (26) NCT00045968	III	232 (232 nd)	99^2^	Glioma stem cell lysate		ID	3x every 10 days, 3x monthly, then every 6 months|2.6 × 10^6^ cells	**19.3 (nd), 13.2 (r)**	

nd, newly diagnosed; r, recurrent; TAA, tumor-associated antigen; SC, subcutaneous; ID, intradermal; IN, intranodal; mOS, median overall survival; PFS, progression free survival; m, months. ^†^Bold indicates statistically significant increase. ^1^Significant only after stratification for IDH1 and TERT promoter mutation status. Additionally, the study does not discriminate between newly diagnosed and recurrent GBM patients when reported survival results. ^2^Sixty four controls crossed over to receive vaccine after tumor recurrence and were analyzed as recurrent GBM patients. External controls were used for statistical analysis.

## 4. Optimization of dendritic cell vaccination

Despite promising data demonstrated by early clinical trials investigating the use of DCV for GBM, additional research is required to further optimize efficacy. There are several possible strategies by which DCV efficacy may be improved. Key strategies include optimal antigen selection, improved vaccine modulation strategies, and enhanced monitoring of treatment response.

### 4.1. Antigen selection

While research examining GBM antigen expression has demonstrated considerable heterogeneity between patients and tumor cytogenetic subtypes, key antigens have shown promise as potential targets for dendritic cell vaccination across multiple GBM patients. For example, Epidermal Growth Factor Receptor variant III (EGFRvIII), a mutant form of EGFR that is constitutively active and highly specific to GBM, is associated with tumor growth and progression. EGFRvIII has been extensively studied as a target for immunotherapy and thus may be an important component of an effective DCV across patients (36–38). Similarly, IL-13Rα2 (Interleukin-13 receptor alpha 2) is rarely expressed in normal brain tissue but overexpressed in 40–60% of GBM cases (39). It is involved in promoting tumor growth and invasion and may also serve as an effective target for DCV. Other key antigens may include Human Epidermal Growth Factor Receptor 2 (HER2), Sex-determining Region Y-box 2 (SOX2), Wilms Tumor 1 (WT1), Melanoma-associated antigen 1 (MAGE-A1), Absent in Melanoma 2 (AIM2), and Tyrosinase-related protein 2 (TRP-2) (36–38).

In addition to these specific antigens, whole tumor cell lysates have also been investigated as a source for dendritic cell vaccination. Utilizing whole tumor lysates offers the advantage of presenting a wider array of tumor-associated antigens to the immune system, addressing the issue of tumor heterogeneity. One recent study investigated the uptake of GBM tumor cell lysates by dendritic cells. Utilizing confocal microscopy, researchers demonstrated that dendritic cells not only internalized, but also effectively presented these tumor antigens in the context of both MHC class I and II molecules. When the lysate-loaded dendritic cells were introduced to T cells, they demonstrated pronounced antitumoral cytotoxic effects (40). A phase I clinical trial (NCT02010606) employing an autologous dendritic cell vaccine pulsed with lysate from a GBM stem-like cell line demonstrated that patients with newly diagnosed GBM had a median overall survival of 20.36 months, while those with recurrent GBM had a median survival of 11.97 months. Moreover, a subset of these patients exhibited a robust cytotoxic T-cell response (41). While recent studies have shown promising results with DC vaccines derived from whole tumor lysates, further research is required to directly compare the efficacy and specificity of the antigen-specific method versus the utilization of tumor cell lysates in DCV. The potential and limitations of this approach require further elucidation in clinical trials.

### 4.2. Immunomodulatory agents

Several immunomodulatory agents have been investigated to activate and enhance the function of DCV and may be effective against GBM, including cytokines and toll-like receptor (TLR) agonists. For instance, IL-2 plays a crucial role in T cell activation and proliferation and has been used in combination with DCV to enhance the expansion and activation of tumor-specific T cells (39). Similarly, GM-CSF promotes the maturation and activation of dendritic cells and has been studied as an adjuvant to enhance the immunostimulatory properties of DCV (42). IL-12 promotes the development of T helper 1 (Th1) immune responses and has been used to augment the antitumor immune response in combination with DCV (43). Thus, identifying additional agents to modulate the function of DCV and stimulate antitumor immunity will be a key strategy for continuing to optimize therapeutic efficacy (Table 2).

**Table 2:** Strategies for optimization of dendritic cell vaccination for malignant gliomas.

Tumor gene targets	Associated protein	Supporting literature
EGFRvIII	Epidermal growth factor receptor variant III	Saikali et al. (44), Sampson et al. (45), An et al. (46)
IL-13Rα2	Interleukin-13 receptor subunit alpha-2	Saikali et al. (44), Jarboe et al. (47), Knudson et al. (48)
HER2	Human epidermal growth factor receptor 2	Wang et al. (38), Ahmed et al. (49), Zhang et al. (50)
SOX2	SRY-Box transcription factor 2	Wang et al. (38), Garros-Regulez et al. (51)
WT1	Wilms tumor 1	Wang et al. (38), Sakai et al. (52), Oji et al. (53)
MAGE-A1	Melanoma-associated antigen 1	Wang et al. (38), Shi et al. (54)
AIM2	Absent in melanoma 2	Chen et al. (36), Liu et al. (55)
TRP-2	Tyrosinase related protein-2	Liu et al. (37), Liu et al. (56), Saikali et al. (44)
Vaccine modulation targets		
IL-2	Interleukin-2	Shimizu et al. (39), Miki et al. (57)
GM-CSF	Granulocyte-macrophage colony-stimulating factor	Li et al. (42), Driessens et al. (58), Zhang et al. (59)
IL-12	Interleukin-12	Homma et al. (43), Kim et al. (60), Giermasz et al. (61)
TLR3/7/8	Toll-like receptors 3, 7, 8	Prins et al. (62), Mehrotra et al. (63), Waele et al. (64)

### 4.3. Utility of FDG-PET

Positron Emission Tomography with 18F-fludeoxyglucose (FDG-PET) visualizes metabolic activity in tissues and may be a valuable resource for both research and clinical applications of DCV for GBM. FDG-PET can be used to assess the response to DCV by monitoring changes in metabolic activity within the tumor. Following vaccination, a reduction in metabolic activity or tumor burden as revealed by FDG-PET may indicate a positive treatment response, while persistent or increased metabolic activity may suggest a lack of response or tumor progression. It is important to note that general immune stimulatory effects might increase the FDG uptake in the immediate period following vaccination. Immune cell infiltrates might increase metabolic responses often indicate treatment response. This phenomenon, known as pseudoprogression, is characterized by an initial appearance of disease progression—manifested by an increase in lesion size and FDG-avidity, or an increase in the number of FDG-avid lesions—within the first 12 weeks of immunotherapy. Importantly, this is subsequently followed by a reduction in tumor burden upon continued administration of immunotherapy (65).

Early detection of immunotherapy-induced tumor response is pivotal, yet it can be confounded by therapy-induced pseudoprogression. The need to modify existing response definitions, as delineated by the Response Evaluation Criteria in Solid Tumors (RECIST), arose primarily from observed pseudoprogression in patients treated with ipilimumab. A consensus guideline, iRECIST, was developed as a modification of Response Evaluation Criteria in Solid Tumors (RECIST) (66). Beyond traditional standardized uptake value (SUV) metrics, leveraging metrics like metabolic active tumor volume (MATV) and total lesion glycolysis (TLG) can provide more comprehensive insights. Specifically, MATV can be viewed as the PET counterpart of iRECIST, offering a holistic assessment of all identified lesions (67, 68). By leveraging FDG-PET heterogeneity parameters, a clearer distinction between pseudoprogression and true progression may be achieved. Pseudoprogressing lesions, influenced by the immune infiltrate, may present unique heterogeneity patterns. Pooling data across centers, while ensuring compatibility in PET reconstruction parameters, can enhance robustness and reproducibility (69). A collaborative approach can facilitate the precise identification of pseudoprogression using advanced PET quantitative measures. Beyond its applications in DCV for GBM, FDG-PET has been explored for tracing treatment responses in other immunotherapeutic modalities. For instance, in melanoma and lung cancer, FDG-PET has shown potential in predicting responses to checkpoint inhibitors, providing early insights into therapeutic outcomes (68). Such findings accentuate the versatility of FDG-PET as a valuable tool across diverse immunotherapeutic strategies.

FDG-PET has been used as a clinical tool for evaluating treatment response to gamma knife therapy in GBM (70–72). In a similar way, regular FDG-PET scans over the course of treatment may be a valuable tool for tracking the efficacy of DCV, leading to an enhanced understanding of response-mechanisms in DCV. Furthermore, FDG-PET can also help identify suitable target lesions for DCV. GBM tumors are known for their intratumoral heterogeneity, with different regions exhibiting varying degrees of aggressiveness and response to treatment (73, 74). “Hot” areas of FDG uptake within the tumor are more metabolically active and are likely to contain important tumor antigens for DCV-targeting. Additional delayed imaging can be utilized to separate inflammatory reactions from tumor viability and progression. Of note, false positives can occur when there is high FDG-PET uptake in normal cortex or local seizure activity, confounding the interpretation of such studies (65).

FDG-PET imaging parameters can also potentially serve as prognostic indicators for GBM patients receiving DCV and thus inform patient selection and treatment strategy. Key parameters measured by FDG-PET include metabolic tumor volume (MTV), TLG, maximum standardized uptake value (SUVmax), and tumor-to-background ratio (TBR). These parameters have been independently studied as valuable prognostic indicators (75–77). By providing valuable information about tumor metabolism, FDG-PET imaging can therefore aid in patient selection, treatment planning, and monitoring the response to DCV for GBM. To enhance the utility of FDG-PET in these cases, FDG-PET scans can also be co-registered with MRI to refine analysis and identify true progression vs. treatment response; one study showed that, in a cohort of 5 patients, 3 showed tumor progression on MRI while showing treatment response with PET; this indicates there may be benefit in dual imaging techniques (78).

In conclusion, FDG-PET imaging can assist with early studies evaluating the efficacy of DCV treatment in conjunction with other accepted therapies. For example, if DCV is combined with chemotherapy or radiation therapy, FDG-PET can assess both the individual and synergistic effects of these treatments on tumor activity. FDG-PET imaging allows clinicians to assess tumor characteristics non-invasively, facilitates personalized treatment approaches and decision-making in the context of dendritic cell vaccination, and may facilitate future research as DCV continues to evolve as a novel immunotherapy for GBM.

## 5. Integration with traditional and emerging therapeutics

Cytoreductive surgery is often conducted before DCV is administered to patients. A correlation between the extent of resection and enhanced survival outcomes has been identified in numerous studies, thereby establishing its predictive value independently. Furthermore, minimal residual disease is speculated to provide benefits in the context of vaccination therapy (33). The perceived advantages can be attributed to the reduction in local immunosuppression, which has a strong correlation with tumor size (28). Additionally, a substantial population of rapidly dividing tumor cells, typically eliminated by cytotoxic T lymphocytes, may contribute to the observed positive effects.

It is essential to clarify that DCV treatment can be effectively employed as an adjunct to the standard of care, which includes temozolomide (TMZ) chemotherapy. While one study reported no significant correlation between the extent of surgical resection and survival rates, the potential benefits of DCV in enhancing the effectiveness of SOC should not be overlooked (28). A comprehensive evaluation is still warranted, which should encompass multiple variables such as total residual tumor volume, tumor composition, and the effects of an immunosuppressive tumor microenvironment. Though various clinical trials have investigated the use of DCV as a standalone treatment, it’s crucial to assess the potential synergistic advantages and disadvantages when DCV is combined with established SOC protocols, including TMZ chemotherapy (79).

In glioma patients, DCs often exhibit diminished functionality or tolerance, not only due to the inhibitory effects imposed by the immune microenvironment on DC proliferation and differentiation but also due to the heterogeneity of GBM molecular subtypes. This heterogeneity is a significant challenge because distinct subtypes possess varied immunophenotypes, potentially leading to differential clinical outcomes. While the immune microenvironment suppresses DC activity, the molecular diversity further complicates the targeted immune response (80).

To address these multifaceted challenges, one approach has been the *in vitro* administration of actively matured DCs, which can trigger the activation of suppressed T cells that migrate into the brain via lymphatic reflux. This mechanism serves as a compensatory method, enhancing the adaptive immune response in patients (79). Moreover, to potentiate DCV’s effectiveness, combination therapies with immune checkpoint inhibitors, such as PD-1/PD-L1 inhibitors, have been explored. Evidence from melanoma treatments supports this combinatorial approach: melanoma patients who experienced recurrence after adjuvant DC vaccination, when treated with first- or second-line PD-1 inhibitor monotherapy, showed a noteworthy response rate of 52% (13, 81).

The therapeutic effects of mature DCs are conveyed through the upregulation of stimulatory receptors such as CD80/86, and the downregulation of inhibitory receptors, including PD-L1 and CTLA-4. Immunodetection indicators frequently used in glioma patients post-treatment include CTLA-4 and PD-L1 (82). A study involving 27 GBM patients who received DCs loaded with tumor antigens revealed that those with a lower PD-1+/CD8+ ratio in their tumor-infiltrating lymphocytes demonstrated prolonged overall survival (OS) and progression-free survival (PFS). DC vaccination substantially reduces PD-1 expression in T cells, thereby improving the tumor microenvironment and enhancing the efficacy of cytotoxic T cells in eradicating tumor cells (33).

Upon antigen loading, DCs intricately regulate the expression of pro-inflammatory cytokines, mitigate negative cytokines, and modulate the migration of other immune cells. This coordination ultimately enhances the body’s anti-tumor immunity and improves the tumor microenvironment (83). Notably, a study involving intratumoral injection of antigen-pulsed DC cells demonstrated enhancements in the tumor microenvironment, characterized by decreased transforming growth factor beta (TGF-β) levels, increased tumor necrosis factor alpha (TNF-α) and IFN-γ levels, facilitated proliferation of CD8+ T cells, reduced activation of Tregs, and improved survival rates in mice with glioma (84).

The integration of DC vaccines with other therapeutic modalities enables the targeting of multiple pathways, thereby addressing immunosuppression within the tumor microenvironment. The current treatment approach for GBM includes surgical resection to decrease tumor burden and prolong survival (85). DC vaccines are then administered concurrently with radiotherapy, chemotherapy, or both, aiming to induce DNA damage and endoplasmic reticulum stress, which ultimately lead to cell death and the release of chemokines and cytokines that augment DC stimulation signals. This combined approach reinforces the anti-tumor effects of DC vaccines. Additionally, specific targeted therapies can be utilized alongside DC activation to obstruct alternative pathways. For instance, targeting the blood–brain barrier facilitates improved drug delivery, while interventions directed toward signaling pathways such as the tumor suppressor genes p53, Rb, and receptor tyrosine kinases (RTK) or the use of cytokines can selectively inhibit myeloid-derived suppressor cells, Tregs, and microglia (86). Notably, the inhibition of CSF-1R using BLZ945 effectively reduces the activity of microglia and the activation of M2 macrophages, thereby enhancing the immune response and median survival. When combined with DC vaccines, this approach presents a promising strategy to reduce immune evasion by tumor cells and offers novel prospects for extending median survival (87).

## 6. Challenges and future perspectives

DCV has been recognized as a promising conduit to exploit the immunological response against glioblastoma. Nevertheless, this arena is fraught with complications and many facets necessitate additional exploration.

The intrinsic heterogeneity of glioblastomas presents a substantial impediment to the efficiency of DCV. As the most aggressive form of brain cancer, glioblastomas are characterized by a high degree of intratumoral and intertumoral variability. The varied genetic and phenotypic attributes inherent to neoplastic cells may trigger disparate immune reactions, consequently influencing the therapeutic potency of the vaccine (88). Another critical hurdle is the immunosuppressive tumor milieu, which might abet the resistance of glioblastoma to DCV treatment. The existence of regulatory T cells (Tregs), myeloid-derived suppressor cells, and molecules such as PD-L1 could potentially debilitate dendritic cell activity and antigen presentation (89). Furthermore, TREM2, known for its elevated expression in myeloid subsets including macrophages and microglia, has been associated with a poor prognosis in glioma. Targeting TREM2 represents a promising strategy to counteract the immunosuppressive environment within the tumor. When paired with DCV, targeting TREM2 could substantially enhance therapeutic outcomes by modulating the tumor microenvironment to be more receptive to immune interventions (90).

The integration of DCV with standard-of-care therapies for glioblastoma presents unique challenges and opportunities. While surgery, radiotherapy, and temozolomide serve as mainstay treatments for glioblastoma, these approaches can substantially influence the immune response, which has direct implications for the effectiveness of DCV. Surgery, the primary therapeutic intervention for glioblastoma, induces a profound stress response that could further impact immune functionality. This could potentiate immunosuppression, possibly constraining the effectiveness of subsequent DCV (16). The timing of DCV administration alongside surgical intervention for the best synergistic effect remains a critical subject of exploration. Another fundamental component of glioblastoma treatment, radiotherapy, can initiate immunogenic cell death, precipitating the liberation of tumor antigens and alarm signals that could potentially amplify DCV effectiveness. Conversely, radiotherapy can also induce lymphopenia and augment the expression of immunosuppressive molecules, such as PD-L1, within the tumor environment, which could thwart DCV functionality. The challenge lies in harnessing the immune-stimulatory effects while mitigating the immune-suppressive effects of radiotherapy (91). The standard chemotherapy for glioblastoma, Temozolomide, also induces lymphopenia and the ensuing immunosuppression could potentially reduce the efficacy of DCVs (92).

DCV manufacturing is also a complex multifaceted procedure involving distinct methods for the generation of dendritic cells, antigen selection, and patient conditioning, factors that could substantially affect the outcome of the treatment (93). The broad spectrum of antigens eligible for loading onto DCVs presents an array of concerns while simultaneously offering intriguing prospects for future exploration. One issue encountered in the context of tumor peptide-loaded DCVs is the pivotal nature of peptide selection, which must accurately reflect the variety of antigens expressed by the tumor. Furthermore, the identification of tumor-specific peptides presents a significant challenge due to the pronounced molecular mimicry between neoplastic and normal cells. The heterogeneity of the tumor further complicates this scenario, as disparate tumor cells may express differing peptide sets, thereby adding more complexity to the peptide selection process (94). Another prevalent strategy involves tumor lysate-loaded DCVs which are not only technically difficult to obtain, but the need for associated immunosuppressive elements may hamper the receiver’s immune response (95). mRNA-loaded DCVs grapple with the issue of the inherent instability of mRNA, which can degrade prior to its delivery to the dendritic cells. This necessitates a concerted effort to ensure compound stability along with an extensive understanding of the tumor antigenic profile (96). The primary obstacle associated with stem cell-loaded DCVs is their inherent heterogeneity and variability in antigen expression, which can result in inconsistent immune responses, thereby impacting the vaccine’s effectiveness (97). Another notable challenges is understanding the variability in patient responses. A patient’s immunophenotype may play a pivotal role in influencing their response to DCV. It would be enlightening to compare vaccine responders and non-responders, as this would shed light on potential markers that could be employed to monitor patient status.

Despite these challenges, there is optimism for the future of DCV in glioblastoma. Further comprehension of neoplastic biology and immunological mechanisms may direct the development of safer and more effective DCV strategies. Harnessing neoantigens, unique to each patient’s tumor, may enhance DCV’s efficacy. Tailor-made DCV methodologies, premised on individual patients’ tumor characteristics, may emerge as a viable modality (18, 98). As the field moves forward, ensuring rigorous DCV quality control is imperative. For instance, determining the precise DC dosage is paramount. An optimal dose ensures that there are enough DCs to instigate the desired immune response. Furthermore, discerning the optimal temporal window—whether aligned with the disease’s progression or the condition of the patient’s immune system—is pivotal for enhancing therapeutic potential. Researchers must optimize schedules and administration routes for therapeutic vaccine protocols (99, 100). Additionally, further research regarding the route of administration is essential to better understand implications for treatment, safety, and efficacy.

Treatments modulating the immune system’s response to standard-of-care therapies may enhance the synergistic effects of DCV. For example, radiotherapy-induced immunogenic cell death can potentially be harnessed to augment DCV efficacy (101). Innovations in the process of DCV production could play a pivotal role in enhancing treatment results. Systematization of protocols, stringent quality checks, and the development of expedited antigen delivery systems are cardinal research trajectories. For instance, the use of nanocarriers can improve the delivery and uptake of DCV, enhancing its ability to stimulate immune cells and induce an anti-tumor response (100). Additionally, with further exploration of its efficacy, imaging, such as FDG-PET, can play a pivotal role in guiding the selection of treatment sites and monitoring response to treatment. In line with this, targeted delivery systems could ensure that the DCV reaches the tumor site, improving its effectiveness and reducing potential systemic side effects.

## 7. Conclusion

While results vary across trials, DCV presents a promising and generally safe treatment strategy for GBM. The complex interactions between standard-of-care therapies and DCVs present both challenges and opportunities for glioblastoma treatment. Importantly, the adaptability of DCV suggests potential applications in the realm of personalized medicine tailored to individual patient needs. Future studies should aim to fully elucidate these interactions in order to optimize the timing, sequencing, and dosage of these treatments when combined with DCV, potentially improving the prognosis for glioblastoma patients. Further research regarding the role of imaging studies in the treatment of glioblastoma may also provide additional insight into assessing tumor burden effectively and accurately. Thus, sustained research endeavors are pivotal to navigating these challenges and unveiling the full potential of DCV for patients diagnosed with glioblastoma.

## Author contributions

RS: Writing – original draft, Writing – review & editing. ET: Writing – original draft, Writing – review & editing. ArA: Writing – original draft, Writing – review & editing. CP: Writing – original draft, Writing – review & editing. ST: Writing – original draft, Writing – review & editing. I-MM: Writing – original draft, Writing – review & editing. AhA: Writing – original draft, Writing – review & editing. VA: Writing – original draft, Writing – review & editing. OS: Writing – original draft, Writing – review & editing. SM: Writing – original draft, Writing – review & editing. AL: Writing – original draft, Writing – review & editing. TW: Supervision, Writing – original draft. M-ER: Supervision, Writing – original draft, Writing – review & editing. AbA: Supervision, Writing – original draft, Writing – review & editing.

## References

[1] Reardon DA Mitchell DA. The development of dendritic cell vaccine-based immunotherapies for glioblastoma. Semin Immunopathol. (2017) 39:225–39. doi: 10.1007/s00281-016-0616-7, PMID: 28138787

[2] Olar A Aldape KD. Using the molecular classification of glioblastoma to inform personalized treatment. J Pathol. (2014) 232:165–77. doi: 10.1002/path.4282, PMID: 24114756 PMC4138801

[3] Tang LW Mallela AN Deng H Richardson TE Hervey-Jumper SL McBrayer SK. Preclinical modeling of lower-grade gliomas. Front Oncol. (2023) 13:1139383. doi: 10.3389/fonc.2023.1139383 37051530 PMC10083350

[4] Rosenblatt J Vasir B Uhl L Blotta S MacNamara C Somaiya P. Vaccination with dendritic cell/tumor fusion cells results in cellular and humoral antitumor immune responses in patients with multiple myeloma. Blood. (2011) 117:393–402. doi: 10.1182/blood-2010-04-277137 21030562 PMC3031474

[5] Sabado RL Bhardwaj N. Dendritic-cell vaccines on the move. Nature. (2015) 519:300–1. doi: 10.1038/nature14211, PMID: 25762139

[6] Cho D-Y Yang W-K Lee H-C Hsu D-M Lin H-L Lin S-Z. Adjuvant immunotherapy with whole-cell lysate dendritic cells vaccine for glioblastoma multiforme: a phase II clinical trial. World Neurosurg. (2012) 77:736–44. doi: 10.1016/j.wneu.2011.08.020, PMID: 22120301

[7] Batich KA Mitchell DA Healy P Herndon JE II Sampson JH. Once, twice, three times a finding: reproducibility of dendritic cell vaccine trials targeting cytomegalovirus in glioblastoma. Clin Cancer Res. (2020) 26:5297–303. doi: 10.1158/1078-0432.CCR-20-1082, PMID: 32719000 PMC9832384

[8] Song L Dong G Guo L Graves DT. The function of dendritic cells in modulating the host response. Mol Oral Microbiol. (2018) 33:13–21. doi: 10.1111/omi.12195, PMID: 28845602 PMC5771978

[9] Merad M Sathe P Helft J Miller J Mortha A. The dendritic cell lineage: ontogeny and function of dendritic cells and their subsets in the steady state and the inflamed setting. Annu Rev Immunol. (2013) 31:563–604. doi: 10.1146/annurev-immunol-020711-074950, PMID: 23516985 PMC3853342

[10] Mellman I. Dendritic cells: master regulators of the immune response. Cancer Immunol Res. (2013) 1:145–9. doi: 10.1158/2326-6066.CIR-13-0102 24777676

[11] Filin IY Kitaeva KV Rutland CS Rizvanov AA Solovyeva VV. Recent advances in experimental dendritic cell vaccines for cancer. Front Oncol. (2021) 11:730824. doi: 10.3389/fonc.2021.730824 34631558 PMC8495208

[12] Calmeiro J Carrascal MA Tavares AR Ferreira DA Gomes C Falcão A. Dendritic cell vaccines for Cancer immunotherapy: the role of human conventional type 1 dendritic cells. Pharmaceutics. (2020) 12:158. doi: 10.3390/pharmaceutics12020158, PMID: 32075343 PMC7076373

[13] Yu J Sun H Cao W Song Y Jiang Z. Research progress on dendritic cell vaccines in cancer immunotherapy. Exp Hematol Oncol. (2022) 11:3. doi: 10.1186/s40164-022-00257-2, PMID: 35074008 PMC8784280

[14] Srivastava S Jackson C Kim T Choi J Lim M. A characterization of dendritic cells and their role in immunotherapy in glioblastoma: from preclinical studies to clinical trials. Cancers. (2019) 11:537. doi: 10.3390/cancers11040537, PMID: 30991681 PMC6521200

[15] Hsu FJ Benike C Fagnoni F Liles TM Czerwinski D Taidi B. Vaccination of patients with B-cell lymphoma using autologous antigen-pulsed dendritic cells. Nat Med. (1996) 2:52–8. doi: 10.1038/nm0196-52, PMID: 8564842

[16] Datsi A Sorg RV. Dendritic cell vaccination of glioblastoma: road to success or dead end. Front Immunol. (2021) 12:770390. doi: 10.3389/fimmu.2021.770390 34795675 PMC8592940

[17] Liau LM Black KL Martin NA Sykes SN Bronstein JM Jouben-Steele L. Treatment of a glioblastoma patient by vaccination with autologous dendritic cells pulsed with allogeneic major histocompatibility complex class I–matched tumor peptides: case report. Neurosurg Focus. (2000) 9:1–5. doi: 10.3171/foc.2000.9.6.9, PMID: 16817691

[18] Eagles ME Nassiri F Badhiwala JH Suppiah S Almenawer SA Zadeh G. Dendritic cell vaccines for high-grade gliomas. Ther Clin Risk Manag. (2018) 14:1299–313. doi: 10.2147/TCRM.S135865, PMID: 30100728 PMC6067774

[19] Liau LM Prins RM Kiertscher SM Odesa SK Kremen TJ Giovannone AJ. Dendritic cell vaccination in glioblastoma patients induces systemic and intracranial T-cell responses modulated by the local central nervous system tumor microenvironment. Clin Cancer Res. (2005) 11:5515–25. doi: 10.1158/1078-0432.CCR-05-0464, PMID: 16061868

[20] Mitchell DA Sayour EJ Reap E Schmittling R DeLeon G Norberg P. Severe adverse immunologic reaction in a patient with glioblastoma receiving autologous dendritic cell vaccines combined with GM-CSF and dose-intensified temozolomide. Cancer Immunol Res. (2015) 3:320–5. doi: 10.1158/2326-6066.cir-14-0100, PMID: 25387895 PMC4510873

[21] Rutkowski S De Vleeschouwer S Kaempgen E Wolff JEA Kühl J Demaerel P. Surgery and adjuvant dendritic cell-based tumour vaccination for patients with relapsed malignant glioma, a feasibility study. Br J Cancer. (2004) 91:1656–62. doi: 10.1038/sj.bjc.6602195, PMID: 15477864 PMC2409960

[22] Jie X Hua L Jiang W Feng F Feng G Hua Z. Clinical application of a dendritic cell vaccine raised against heat-shocked glioblastoma. Cell Biochem Biophys. (2012) 62:91–9. doi: 10.1007/s12013-011-9265-6 21909820

[23] Yao Y Luo F Tang C Chen D Qin Z Hua W. Molecular subgroups and B7-H4 expression levels predict responses to dendritic cell vaccines in glioblastoma: an exploratory randomized phase II clinical trial. Cancer Immunol Immunother. (2018) 67:1777–88. doi: 10.1007/s00262-018-2232-y, PMID: 30159779 PMC11028057

[24] Mitchell DA Batich KA Gunn MD Huang M-N Sanchez-Perez L Nair SK. Tetanus toxoid and CCL3 improve dendritic cell vaccines in mice and glioblastoma patients. Nature. (2015) 519:366–9. doi: 10.1038/nature14320, PMID: 25762141 PMC4510871

[25] Batich KA Reap EA Archer GE Sanchez-Perez L Nair SK Schmittling RJ. Long-term survival in glioblastoma with cytomegalovirus pp65-targeted vaccination. Clin Cancer Res. (2017) 23:1898–909. doi: 10.1158/1078-0432.CCR-16-2057, PMID: 28411277 PMC5559300

[26] Liau LM Ashkan K Brem S Campian JL Trusheim JE Iwamoto FM. Association of autologous tumor lysate-loaded dendritic cell vaccination with extension of survival among patients with newly diagnosed and recurrent glioblastoma: a phase 3 prospective externally controlled cohort trial. JAMA Oncol. (2023) 9:112–21. doi: 10.1001/jamaoncol.2022.5370 36394838 PMC9673026

[27] Wen PY Reardon DA Armstrong TS Phuphanich S Aiken RD Landolfi JC. A randomized double-blind placebo-controlled phase II trial of dendritic cell vaccine ICT-107 in newly diagnosed patients with glioblastoma. Clin Cancer Res. (2019) 25:5799–807. doi: 10.1158/1078-0432.CCR-19-0261, PMID: 31320597 PMC8132111

[28] Buchroithner J Erhart F Pichler J Widhalm G Preusser M Stockhammer G. Audencel immunotherapy based on dendritic cells has no effect on overall and progression-free survival in newly diagnosed glioblastoma: a phase II randomized trial. Cancers. (2018) 10:372. doi: 10.3390/cancers10100372, PMID: 30301187 PMC6210090

[29] De Vleeschouwer S. Vaccines against glioblastoma: reflections on the ICT-107 phase IIb trial. Transl Cancer Res. (2020) 9:4473–5. doi: 10.21037/tcr-2020-004, PMID: 35117812 PMC8799264

[30] Aarntzen EHJG Srinivas M Bonetto F Cruz LJ Verdijk P Schreibelt G. Targeting of 111In-labeled dendritic cell human vaccines improved by reducing number of cells. Clin Cancer Res. (2013) 19:1525–33. doi: 10.1158/1078-0432.CCR-12-1879, PMID: 23382117

[31] Wheeler CJ Black KL Liu G Mazer M Zhang X Pepkowitz S. Vaccination elicits correlated immune and clinical responses in glioblastoma multiforme patients. Cancer Res. (2008) 68:5955–64. doi: 10.1158/0008-5472.CAN-07-5973 18632651

[32] Tian Y Liu C Li Z Ai M Wang B Du K. Exosomal B7–H4 from irradiated glioblastoma cells contributes to increase FoxP3 expression of differentiating Th1 cells and promotes tumor growth. Redox Biol. (2022) 56:102454. doi: 10.1016/j.redox.2022.102454, PMID: 36044789 PMC9440073

[33] Jan C-I Tsai W-C Harn H-J Shyu W-C Liu M-C Lu H-M. Predictors of response to autologous dendritic cell therapy in glioblastoma Multiforme. Front Immunol. (2018) 9:727. doi: 10.3389/fimmu.2018.00727, PMID: 29910795 PMC5992384

[34] Prins RM Wang X Soto H Young E Lisiero DN Fong B. Comparison of glioma-associated antigen peptide-loaded versus autologous tumor lysate-loaded dendritic cell vaccination in malignant glioma patients. J Immunother. (2013) 36:152–7. doi: 10.1097/CJI.0b013e3182811ae4, PMID: 23377664 PMC3568250

[35] Fong B Jin R Wang X Safaee M Lisiero DN Yang I. Monitoring of regulatory T cell frequencies and expression of CTLA-4 on T cells, before and after DC vaccination, can predict survival in GBM patients. PLoS One. (2012) 7:e32614. doi: 10.1371/journal.pone.0032614, PMID: 22485134 PMC3317661

[36] Chen PA Shrivastava G Balcom EF McKenzie BA Fernandes J Branton WG. Absent in melanoma 2 regulates tumor cell proliferation in glioblastoma multiforme. J Neuro-Oncol. (2019) 144:265–73. doi: 10.1007/s11060-019-03230-y, PMID: 31280432

[37] Liu G Akasaki Y Khong HT Wheeler CJ Das A Black KL. Cytotoxic T cell targeting of TRP-2 sensitizes human malignant glioma to chemotherapy. Oncogene. (2005) 24:5226–34. doi: 10.1038/sj.onc.1208519, PMID: 15897911

[38] Wang Q-T Nie Y Sun S-N Lin T Han R-J Jiang J. Tumor-associated antigen-based personalized dendritic cell vaccine in solid tumor patients. Cancer Immunol Immunother. (2020) 69:1375–87. doi: 10.1007/s00262-020-02496-w 32078016 PMC11027674

[39] Shimizu K Fields RC Giedlin M Mulé JJ. Systemic administration of interleukin 2 enhances the therapeutic efficacy of dendritic cell-based tumor vaccines. Proc Natl Acad Sci. (1999) 96:2268–73. doi: 10.1073/pnas.96.5.2268, PMID: 10051630 PMC26772

[40] De Vleeschouwer S Arredouani M Adé M Cadot P Vermassen E JanL C. Uptake and presentation of malignant glioma tumor cell lysates by monocyte-derived dendritic cells. Cancer Immunol Immunother. (2005) 54:372–82. doi: 10.1007/s00262-004-0615-8, PMID: 15692847 PMC11042490

[41] Hu JL Omofoye OA Rudnick JD Kim S Tighiouart M Phuphanich S. A phase I study of autologous dendritic cell vaccine pulsed with allogeneic stem-like cell line lysate in patients with newly diagnosed or recurrent glioblastoma. Clin Cancer Res. (2021) 28:689–96. doi: 10.1158/1078-0432.CCR-21-2867 34862245

[42] Li M Wang B Wu Z Zhang J Shi X Cheng W. A novel recombinant protein of ephrinA1–PE38/GM-CSF activate dendritic cells vaccine in rats with glioma. Tumor Biol. (2015) 36:5497–503. doi: 10.1007/s13277-015-3217-5, PMID: 25677907

[43] Homma S Komita H Sagawa Y Ohno T Toda G. Antitumour activity mediated by CD4+ cytotoxic T lymphocytes against MHC class II-negative mouse hepatocellular carcinoma induced by dendritic cell vaccine and interleukin-12. Immunology. (2005) 115:451–61. doi: 10.1111/j.1365-2567.2005.02179.x, PMID: 16011514 PMC1782174

[44] Saikali S Avril T Collet B Hamlat A Bansard J-Y Drenou B. Expression of nine tumour antigens in a series of human glioblastoma multiforme: interest of EGFRvIII, IL-13Rα2, gp100 and TRP-2 for immunotherapy. J Neurooncol (2007) 81:139–48. doi: 10.1007/s11060-006-9220-3 17004103

[45] Sampson JH Archer GE Mitchell DA Heimberger AB Bigner DD. Tumor-specific immunotherapy targeting the EGFRvIII mutation in patients with malignant glioma. Semin Immunol (2008) 20:267–75. doi: 10.1016/j.smim.2008.04.001 18539480 PMC2633865

[46] An Z Aksoy O Zheng T Fan Q-W Weiss WA. Epidermal growth factor receptor and EGFRvIII in glioblastoma: signaling pathways and targeted therapies. Oncogene (2018) 37:1561–75. doi: 10.1038/s41388-017-0045-7 29321659 PMC5860944

[47] Jarboe JS Johnson KR Choi Y Lonser RR Park JK. Expression of interleukin-13 receptor alpha2 in glioblastoma multiforme: implications for targeted therapies. Cancer Res (2007) 67:7983–6. doi: 10.1158/0008-5472.CAN-07-1493 17804706

[48] Knudson KM Hwang S McCann MS Joshi BH Husain SR Puri RK. Recent Advances in IL-13Rα2-Directed Cancer Immunotherapy. Frontiers in Immunology (2022) 13. Available at: https://www.frontiersin.org/articles/10.3389/fimmu.2022.878365 10.3389/fimmu.2022.878365 PMC9023787 35464460

[49] Ahmed N Salsman VS Kew Y Shaffer D Powell S Zhang YJ. HER2-Specific T Cells Target Primary Glioblastoma Stem Cells and Induce Regression of Autologous Experimental Tumors. Clin. Cancer Res. (2010) 16:474–85. doi: 10.1158/1078-0432.CCR-09-1322 20068073 PMC3682507

[50] Zhang C Burger MC Jennewein L Genßler S Schönfeld K Zeiner P. ErbB2/HER2-Specific NK Cells for Targeted Therapy of Glioblastoma. JNCI: J. Natl. Cancer Inst. (2016) 108:djv375. doi: 10.1093/jnci/djv375 26640245

[51] Garros-Regulez L Garcia I Carrasco-Garcia E Lantero A Aldaz P Moreno-Cugnon L. Targeting SOX2 as a Therapeutic Strategy in Glioblastoma. Front Oncol. (2016) 6: Available at: https://www.frontiersin.org/articles/10.3389/fonc.2016.00222 27822457 10.3389/fonc.2016.00222 PMC5075570

[52] Sakai K Shimodaira S Maejima S Udagawa N Sano K Higuchi Y. Dendritic cell–based immunotherapy targeting Wilms’ tumor 1 in patients with recurrent malignant glioma. J. Neurosurg. (2015) 123:989–97. doi: 10.3171/2015.1.JNS141554 26252465

[53] Oji Y Hashimoto N Tsuboi A Murakami Y Iwai M Kagawa N. Association of WT1 IgG antibody against WT1 peptide with prolonged survival in glioblastoma multiforme patients vaccinated with WT1 peptide. Int. J. Cancer. (2016) 139:1391–401. doi: 10.1002/ijc.30182 27170523 PMC5089562

[54] Shi H Jiang X Fu P Zhou Y Lu X. Use of dentritic cells pulsed with HLA-A2-restricted MAGE-A1 peptide to generate cytotoxic T lymphocytes against malignant glioma. J Huazhong Univ Sci Technol [Med Sci] (2010) 30:678–82. doi: 10.1007/s11596-010-0564-8 21063856

[55] Liu G Khong HT Wheeler CJ Yu JS Black KL Ying H. Molecular and Functional Analysis of Tyrosinase-Related Protein (TRP)-2 as a Cytotoxic T Lymphocyte Target in Patients With Malignant Glioma. J. Immunother. (2003) 26:301. 12843792 10.1097/00002371-200307000-00002

[56] Liu G Yu JS Zeng G Yin D Xie D Black KL. AIM-2: A Novel Tumor Antigen is Expressed and Presented by Human Glioma Cells. J. Immunother. (2004) 27:220. 15076139 10.1097/00002371-200405000-00006

[57] Miki K Nagaoka K Harada M Hayashi T Jinguji H Kato Y. Combination therapy with dendritic cell vaccine and IL-2 encapsulating polymeric micelles enhances intra-tumoral accumulation of antigen-specific CTLs. Int Immunopharmacol. (2014) 23:499–504. doi: 10.1016/j.intimp.2014.09.025 25284343

[58] Driessens G Nuttin L Gras A Maetens J Mievis S Schoore M. Development of a successful antitumor therapeutic model combining in vivo dendritic cell vaccination with tumor irradiation and intratumoral GM-CSF delivery. Cancer Immunol Immunother (2011) 60:273–81. doi: 10.1007/s00262-010-0941-y 21076828 PMC11029469

[59] Zhang S-N Choi I-K Huang J-H Yoo J-Y Choi K-J Yun C-O. Optimizing DC vaccination by combination with oncolytic adenovirus coexpressing IL-12 and GM-CSF. Mol Ther (2011) 19:1558–68. doi: 10.1038/mt.2011.29 21468000 PMC3149171

[60] Kim C-H Hong M-J Park S-D Kim C-K Park M-Y Sohn H-J. Enhancement of anti-tumor immunity specific to murine glioma by vaccination with tumor cell lysate-pulsed dendritic cells engineered to produce interleukin-12. Cancer Immunol Immunother (2006) 55:1309–19. doi: 10.1007/s00262-006-0134-x 16463038 PMC11029860

[61] Giermasz AS Urban JA Nakamura Y Watchmaker P Cumberland RL Gooding W. Type-1 polarized dendritic cells primed for high IL-12 production show enhanced activity as cancer vaccines. Cancer Immunol Immunother (2009) 58:1329–36. doi: 10.1007/s00262-008-0648-5 19156413 PMC2907477

[62] Prins RM Craft N Bruhn KW Khan-Farooqi H Koya RC Stripecke R. The TLR-7 Agonist, Imiquimod, Enhances Dendritic Cell Survival and Promotes Tumor Antigen-Specific T Cell Priming: Relation to Central Nervous System Antitumor Immunity1. J. Immunol. (2006) 176:157–64. doi: 10.4049/jimmunol.176.1.157 16365406

[63] Mehrotra S Britten CD Chin S Garrett-Mayer E Cloud CA Li. Vaccination with poly(IC:LC) and peptide-pulsed autologous dendritic cells in patients with pancreatic cancer. J. Hematol. Oncol. (2017) 10:82. doi: 10.1186/s13045-017-0459-2 28388966 PMC5384142

[64] De Waele J Verhezen T van der Heijden S Berneman ZN Peeters M Lardon F. A systematic review on poly(I:C) and poly-ICLC in glioblastoma: adjuvants coordinating the unlocking of immunotherapy. J. Exp. Clin. Cancer Res. (2021) 40:213. doi: 10.1186/s13046-021-02017-2 34172082 PMC8229304

[65] Cherk MH Nadebaum DP Barber TW Beech P Haydon A Yap KS. 18F-FDG PET/CT features of immune-related adverse events and pitfalls following immunotherapy. J Med Imaging Radiat Oncol. (2022) 66:483–94. doi: 10.1111/1754-9485.13390, PMID: 35191204 PMC9303622

[66] Seymour L Bogaerts J Perrone A Ford R Schwartz LH Mandrekar S. iRECIST: guidelines for response criteria for use in trials testing immunotherapeutics. Lancet Oncol. (2017) 18:e143–52. doi: 10.1016/S1470-2045(17)30074-8, PMID: 28271869 PMC5648544

[67] Garralda E Laurie SA Seymour L de Vries EGE. Towards evidence-based response criteria for cancer immunotherapy. Nat Commun. (2023) 14:3001. doi: 10.1038/s41467-023-38837-3, PMID: 37225715 PMC10209139

[68] Aide N Hicks RJ Le Tourneau C Lheureux S Fanti S Lopci E. FDG PET/CT for assessing tumour response to immunotherapy. Eur J Nucl Med Mol Imaging. (2019) 46:238–50. doi: 10.1007/s00259-018-4171-4, PMID: 30291373 PMC6267687

[69] Sachpekidis C Kopp-Schneider A Pan L Papamichail D Haberkorn U Hassel JC. Interim [18F]FDG PET/CT can predict response to anti-PD-1 treatment in metastatic melanoma. Eur J Nucl Med Mol Imaging. (2021) 48:1932–43. doi: 10.1007/s00259-020-05137-7, PMID: 33336264 PMC8113306

[70] Chao ST Suh JH Raja S Lee SY Barnett G. The sensitivity and specificity of FDG PET in distinguishing recurrent brain tumor from radionecrosis in patients treated with stereotactic radiosurgery. Int J Cancer. (2001) 96:191–7. doi: 10.1002/ijc.1016, PMID: 11410888

[71] Leiva-Salinas C Muttikkal TJE Flors L Puig J Wintermark M Patrie JT. FDG PET/MRI coregistration helps predict response to gamma knife radiosurgery in patients with brain metastases. AJR Am J Roentgenol. (2019) 212:425–30. doi: 10.2214/AJR.18.20006 30422717

[72] Lee J-K Liu R-S Shiang H-R Pan DH-C. Usefulness of Semiquantitative FDG-PET in the prediction of brain tumor treatment response to gamma knife radiosurgery. J Comput Assist Tomogr. (2003) 27:525–9. doi: 10.1097/00004728-200307000-00012, PMID: 12886136

[73] Inda M-M Bonavia R Seoane J. Glioblastoma Multiforme: a look inside its heterogeneous nature. Cancers. (2014) 6:226–39. doi: 10.3390/cancers6010226, PMID: 24473088 PMC3980595

[74] Becker AP Sells BE Haque SJ Chakravarti A. Tumor heterogeneity in glioblastomas: from light microscopy to molecular pathology. Cancers. (2021) 13:761. doi: 10.3390/cancers13040761, PMID: 33673104 PMC7918815

[75] O JH Choi WH Han EJ Choi E-K Chae BJ Park Y-G. The prognostic value of 18F-FDG PET/CT for early recurrence in operable breast Cancer: comparison with TNM stage. Nucl Med Mol Imaging. (2013) 47:263–7. doi: 10.1007/s13139-013-0232-6, PMID: 24900122 PMC4035170

[76] Yoo J Choi JY Moon SH Bae DS Park SB Choe YS. Prognostic significance of volume-based metabolic parameters in uterine cervical cancer determined using 18F-fluorodeoxyglucose positron emission tomography. Int J Gynecol Cancer. (2012) 22:1226–33. doi: 10.1097/IGC.0b013e318260a905 22810970

[77] Im H-J Bradshaw T Solaiyappan M Cho SY. Current methods to define metabolic tumor volume in positron emission tomography: which one is better? Nucl Med Mol Imaging. (2018) 52:5–15. doi: 10.1007/s13139-017-0493-6, PMID: 29391907 PMC5777960

[78] Kristin Schmitz A Sorg RV Stoffels G Grauer OM Galldiks N Steiger H-J. Diagnostic impact of additional O-(2-[18F]fluoroethyl)-L-tyrosine (18F-FET) PET following immunotherapy with dendritic cell vaccination in glioblastoma patients. Br J Neurosurg. (2021) 35:736–42. doi: 10.1080/02688697.2019.1639615, PMID: 31407920

[79] Stupp R Mason WP van den Bent MJ Weller M Fisher B Taphoorn MJB. Radiotherapy plus concomitant and adjuvant temozolomide for glioblastoma. N Engl J Med. (2005) 352:987–96. doi: 10.1056/NEJMoa043330 15758009

[80] Verhaak RGW Hoadley KA Purdom E Wang V Qi Y Wilkerson MD. Integrated genomic analysis identifies clinically relevant subtypes of glioblastoma characterized by abnormalities in PDGFRA, IDH1, EGFR, and NF1. Cancer Cell. (2010) 17:98–110. doi: 10.1016/j.ccr.2009.12.020, PMID: 20129251 PMC2818769

[81] van Willigen WW Bloemendal M Boers-Sonderen MJ de Groot JWB Koornstra RHT van der Veldt AAM. Response and survival of metastatic melanoma patients treated with immune checkpoint inhibition for recurrent disease on adjuvant dendritic cell vaccination. Onco Targets Ther. (2020) 9:1738814. doi: 10.1080/2162402X.2020.1738814, PMID: 33457087 PMC7790511

[82] Goenka A Tiek D Song X Huang T Hu B Cheng S-Y. The many facets of therapy resistance and tumor recurrence in glioblastoma. Cells. (2021) 10:484. doi: 10.3390/cells10030484, PMID: 33668200 PMC7995978

[83] Zanna MY Yasmin AR Omar AR Arshad SS Mariatulqabtiah AR Nur-Fazila SH. Review of dendritic cells, their role in clinical immunology, and distribution in various animal species. Int J Mol Sci. (2021) 22:8044. doi: 10.3390/ijms22158044, PMID: 34360810 PMC8348663

[84] Pellegatta S Poliani PL Stucchi E Corno D Colombo CA Orzan F. Intra-tumoral dendritic cells increase efficacy of peripheral vaccination by modulation of glioma microenvironment. Neuro Oncol. (2010) 12:377–88. doi: 10.1093/neuonc/nop024, PMID: 20308315 PMC2940598

[85] King JL Benhabbour SR. Glioblastoma Multiforme-a look at the past and a glance at the future. Pharmaceutics. (2021) 13:1053. doi: 10.3390/pharmaceutics13071053, PMID: 34371744 PMC8309001

[86] Nguyen H-M Guz-Montgomery K Lowe DB Saha D. Pathogenetic features and current management of glioblastoma. Cancers. (2021) 13:856. doi: 10.3390/cancers13040856, PMID: 33670551 PMC7922739

[87] Li L Zhou J Dong X Liao Q Zhou D Zhou Y. Dendritic cell vaccines for glioblastoma fail to complete clinical translation: bottlenecks and potential countermeasures. Int Immunopharmacol. (2022) 109:108929. doi: 10.1016/j.intimp.2022.108929, PMID: 35700581

[88] Shireman JM Gonugunta N Zhao L Pattnaik A Distler E Her S. GM-CSF and IL-7 fusion cytokine engineered tumor vaccine generates long-term Th-17 memory cells and increases overall survival in aged syngeneic mouse models of glioblastoma. Aging Cell. (2023) 22:e13864. doi: 10.1111/acel.13864, PMID: 37165998 PMC10352573

[89] Lindau D Gielen P Kroesen M Wesseling P Adema GJ. The immunosuppressive tumour network: myeloid-derived suppressor cells, regulatory T cells and natural killer T cells. Immunology. (2013) 138:105–15. doi: 10.1111/imm.12036, PMID: 23216602 PMC3575763

[90] Yu M Chang Y Zhai Y Pang B Wang P Li G. TREM2 is associated with tumor immunity and implies poor prognosis in glioma. Front Immunol. (2023) 13:1089266. doi: 10.3389/fimmu.2022.1089266, PMID: 36713360 PMC9874686

[91] Inogés S Tejada S de Cerio AL-D Gállego Pérez-Larraya J Espinós J Idoate MA. A phase II trial of autologous dendritic cell vaccination and radiochemotherapy following fluorescence-guided surgery in newly diagnosed glioblastoma patients. J Transl Med. (2017) 15:104. doi: 10.1186/s12967-017-1202-z, PMID: 28499389 PMC5427614

[92] Hunn MK Bauer E Wood CE Gasser O Dzhelali M Ancelet LR. Dendritic cell vaccination combined with temozolomide retreatment: results of a phase I trial in patients with recurrent glioblastoma multiforme. J Neuro Oncol. (2015) 121:319–29. doi: 10.1007/s11060-014-1635-7, PMID: 25366363

[93] Karami Fath M Babakhaniyan K Anjomrooz M Jalalifar M Alizadeh SD Pourghasem Z. Recent advances in glioma Cancer treatment: conventional and epigenetic realms. Vaccines. (2022) 10:1448. doi: 10.3390/vaccines10091448, PMID: 36146527 PMC9501259

[94] Niedbała M Malarz K Sharma G Kramer-Marek G Kaspera W. Glioblastoma: pitfalls and opportunities of immunotherapeutic combinations. Onco Targets Ther. (2022) 15:437–68. doi: 10.2147/OTT.S215997, PMID: 35509452 PMC9060812

[95] Erhart F Buchroithner J Reitermaier R Fischhuber K Klingenbrunner S Sloma I. Immunological analysis of phase II glioblastoma dendritic cell vaccine (Audencel) trial: immune system characteristics influence outcome and Audencel up-regulates Th1-related immunovariables. Acta Neuropathol Commun. (2018) 6:135. doi: 10.1186/s40478-018-0621-2, PMID: 30518425 PMC6280511

[96] Do AS-MS Amano T Edwards LA Zhang L Peralta-Venturina MD Yu JS. CD133 mRNA-loaded dendritic cell vaccination abrogates glioma stem cell propagation in humanized glioblastoma mouse model. Mol Ther. (2020) 18:295–303. doi: 10.1016/j.omto.2020.06.019, PMID: 32728617 PMC7378271

[97] Dillman RO Nistor GI Keirstead HS. Autologous dendritic cells loaded with antigens from self-renewing autologous tumor cells as patient-specific therapeutic cancer vaccines. Hum Vaccin Immunother. (2023) 19:2198467. doi: 10.1080/21645515.2023.2198467 37133853 PMC10294766

[98] Abbasi J. Personalized Cancer vaccine approach safe in early trial. JAMA. (2021) 325:1825. doi: 10.1001/jama.2021.6828, PMID: 33974034

[99] Pombo Antunes AR Scheyltjens I Duerinck J Neyns B Movahedi K Van Ginderachter JA. Understanding the glioblastoma immune microenvironment as basis for the development of new immunotherapeutic strategies. Elife. (2020) 9:e52176. doi: 10.7554/eLife.52176, PMID: 32014107 PMC7000215

[100] Saadeldin MK Abdel-Aziz AK Abdellatif A. Dendritic cell vaccine immunotherapy; the beginning of the end of cancer and COVID-19, a hypothesis. Med Hypotheses. (2021) 146:110365. doi: 10.1016/j.mehy.2020.110365, PMID: 33221134 PMC7836805

[101] Asija S Chatterjee A Yadav S Chekuri G Karulkar A Jaiswal AK. Combinatorial approaches to effective therapy in glioblastoma (GBM): current status and what the future holds. Int Rev Immunol. (2022) 41:582–605. doi: 10.1080/08830185.2022.2101647, PMID: 35938932

